# The antimicrobial efficacy of graphene oxide, double antibiotic paste, and their combination against *Enterococcus faecalis* in the root canal treatment

**DOI:** 10.1186/s12903-023-02718-4

**Published:** 2023-01-13

**Authors:** Fateme Eskandari, Abbas Abbaszadegan, Ahmad Gholami, Yasamin Ghahramani

**Affiliations:** 1 School of Dentistry, Shiraz University of Medical Sciences, Shiraz, Iran; 2 Department of Endodontics, School of Dentistry, Shiraz University of Medical Sciences, Ghasrdasht Street, Shiraz, 71956-15878 Iran; 3 Biotechnology Research Center, Shiraz University of Medical Sciences, Shiraz, Iran; 4 Pharmaceutical Sciences Research Center, Shiraz University of Medical Sciences, Shiraz, Iran; 5 Department of Pharmaceutical Biotechnology, School of Pharmacy, Shiraz University of Medical Sciences, Shiraz, Iran

**Keywords:** Antibacterial, Endodontics, *Enterococcus faecalis*, Graphene oxide, Root canal therapy

## Abstract

**Background:**

Inter-appointment medication of the root canals with appropriate intracanal medicaments has been advocated to improve root canal disinfection. Graphene oxide (GO) has shown promising antimicrobial activity against a wide range of microorganisms, besides the capability of carrying antibiotics. The current study aimed to compare the antibacterial activity of double antibiotic paste (DAP) and GO per se and in combination (GO-DAP) against *Enterococcus faecalis* (*E. faecalis*).

**Methods:**

A total of 108 extracted human mandibular premolars were contaminated with three-week-old *E. faecalis* and subjected to a primary microbial assessment. The samples were categorized into 15 groups concerning the intracanal medicament (DAP, GO, GO-DAP, and control) and contact time (1, 7, and 14 days). Then, the root canals were medicated, incubated, and resubjected to a secondary antimicrobial evaluation. The colony-forming units (CFU) were counted to calculate the antimicrobial efficacy. The data were analyzed via the Kruskal–Wallis test (α = 0.05).

**Results:**

GO-DAP was the only medicament that completely eradicated *E. faecalis* in 1 day. The percentage reduction of CFU/ml in the GO-DAP and DAP groups was higher than that in the GO group at all allocated contact times. Furthermore, a significant decrease of the CFU/ml was seen in the GO and DAP groups after 7 and 14 days of being medicated (*P* < 0.05).

**Conclusion:**

Since GO-DAP improved root canal disinfection, this novel material can be introduced as a promising intracanal medicament against *E. faecalis* even in the short run.

## Introduction

Removal of microorganisms and their by-products from the root canals is the supreme objective of endodontic therapy [1, 2], which is impeded by anatomical complexities like lateral canals, dentinal tubules, and isthmuses [3, 4]. This goal can be best achieved by combining mechanical preparation with different irrigants and intracanal medicaments [5, 6] like calcium hydroxide, triple antibiotic paste (TAP), and double antibiotic paste (DAP), which are commonly used between the treatment sessions [1, 7, 8].

TAP (metronidazole, ciprofloxacin, and minocycline) is efficient against gram-positive, gram-negative, and anaerobic bacteria; however, its minocycline content may induce tooth discoloration [9]. This content is excluded in DAP (metronidazole and ciprofloxacin), which is still adequately efficient against *E. faecalis* and *Porphyromonas gingivalis* [9, 10], and indicated in case of failure of commonly-used medicaments [1]. However, while even short-term application of antibiotic combinations (as in TAP and DAP) can increase the risk of antibacterial resistance, long-term exposure to DAP may cause demineralization and negatively affect the mechanical properties of dental hard tissues [11].

Graphene is a two-dimensional monolayer of single carbon atoms with a honeycomb structure, whose outstanding properties have attracted researchers’ attention [12–14]. Although the exact mechanism of the broad antibacterial activity of graphene-based materials is still unclear, it is generally attributed to several modes of action like the disruption and entrapment of the bacterial cell membrane, annihilator extraction of phospholipid molecules, oxidative stress, and self-killing effect [15, 16].

Graphene oxide (GO) possesses a monolayer of atoms with epoxide, carboxylic acid, and hydroxyl groups on the surface, which results in satisfactory water-solubility and creates a wide surface for pharmaceutical incorporation and group functionalization for specific use [17, 18]. It is a favorable carrier of pharmaceuticals and biomolecules and enhances the mechanical performance and bioactivity of biomaterials [19]. This cost-effective antibacterial material is physically and chemically bactericidal and only mildly cytotoxic to mammalian cells in low doses, causes less bacterial resistance, interacts with pharmaceuticals, and easily modifies surfaces with desired functionalities [20–22]. GO has antimicrobial potential against gram-negative and gram-positive microorganisms [23] and is bactericidal on most common dental pathogenic microorganisms [24].

To promote endodontic antimicrobial agents, efforts have been focused on devising brand-new antibacterial delivery techniques like incorporating nanomaterials [25]. Nanosilver graphene oxide and graphene silver nanoparticles exhibited remarkable antibacterial potency as endodontic irrigants [26, 27]. Moreover, graphene nanoplates were reported as a novel biocompatible material for root canal obturation with improved adhesion and antibacterial efficacy [21]. GO has been recommended as an effective antibiotic carrier [28]. To the best of the authors’ knowledge, graphene and its derivatives have not been investigated as intracanal medicaments. This study aimed to compare the antibacterial efficacy of DAP and GO, per se and in combination, against *E. faecalis*. The null hypothesis was that these medicaments would not significantly reduce the CFU/ml of *E. faecalis* in the root canals.

## Materials and methods

### Materials

Graphite powder was purchased from Tanfeng Graphene Technology Co., Ltd., Jiangsu, China. Phosphoric acid (H_3_PO_4_), sulfuric acid (H_2_SO_4_), potassium permanganate (KMnO_4_), hydrogen peroxide (H_2_O_2_), Phosphate buffer saline (PBS, 10 × concentrate, BioPerformance Certified, suitable for cell culture, pH: 7.4), and hydrochloric acid were purchased from Sigma-Aldrich, Gillingham, UK. Ciprofloxacin and metronidazole were purchased from Alborz Darou, Qazvin, Iran. Ampicillin was purchased from Merck, Darmstadt, Germany. Mueller–Hinton broth (MHB) medium was bought from HiMedia (Mumbai, India) and double distilled water was used in each experiment. All other chemicals including 2.5% and 5.25% NaOCl were purchased from Sigma-Aldrich Corporation, St Louis, MO, USA. EDTA was purchased from Wizard; Rehber Chemical Co., Ltd, Istanbul, Turkey. K-file, ProTaper rotary system, hand pluggers, and spiral filler were purchased from Dentsply, Maillefer, Ballaigues, Switzerland. The 30-gauge needle was purchased from Cerkamed, Poland. Ethylene oxide was purchased from Acecil, Campinas, São Paulo, Brazil. Paper points were purchased from Gapadent Co. Ltd., Korea.

The study design was approved by the Ethics Committee of Shiraz University of Medical Sciences, Shiraz, Iran (IR.SUMS.DENTAL.REC.1400.021). It was conducted in full accordance with ethical principles including the World Medical Association Declaration of Helsinki (version 2008).

### Preparation of the medicaments

Every single step of this study was conducted under strict aseptic conditions. For the preparation of DAP, 500 mg of metronidazole was combined with 500 mg of ciprofloxacin in equal proportions (1:1) [1, 29].

GO was synthesized from graphite flakes through a modified Hummer’s method [30] as follows.

**Fig. 1:**
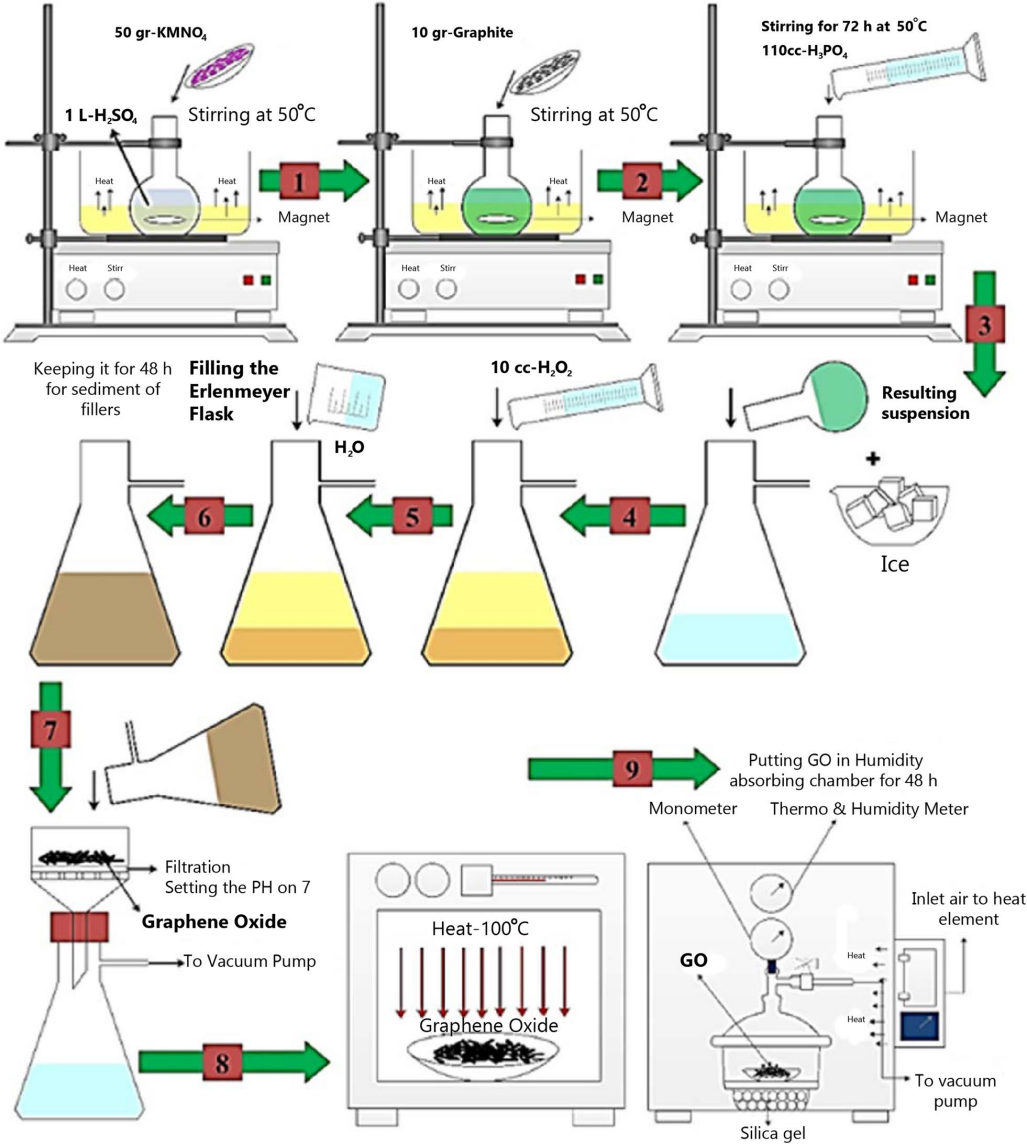
Schematic representation of the synthesis of graphene oxide using modified Hummer’s method. H_3_PO_4_, Phosphoric acid; H_2_SO_4_, Sulfuric acid; KMnO_4_, Potassium permanganate; H_2_O_2_, Hydrogen peroxide

Ten grams of graphite flakes and 110 cc of H_3_PO_4_ and 1 L of H_2_SO_4_ (98%) were mixed in a 1000-ml volumetric flask in an ice bath (0–6 °C) while continually stirred for 2 h. Meanwhile, 50 g of KMnO_4_ was gradually added to the suspension and the addition rate was precisely controlled to maintain the reaction temperature below 14 °C. Then, the ice bath was eliminated and the mixture was stirred at 30 °C till achieving a brown paste and kept under stirring for 2 h. The temperature was raised to 50 °C every half an hour. It was weakened by slowly adding 100 ml of water. The reaction temperature was quickly raised to 96 °C via effervescence and the color went brown. The solution was weakened by adding 100 ml of water while continuously stirred, and eventually treated with 10 ml of H_2_O_2_ to terminate the reaction by the formation of yellow color. For filtration, a Büchner funnel and Whatman filter paper were applied and the Erlenmeyer flask was connected to a vacuum pump. For purification, the mixture was washed by centrifugation and rinsed with 8% HCL, and then deionized water several times. After filtration, it was dried in a hot air oven at 100 °C and later in a humidity-absorbing chamber for 48 h, the GO was attained as a powder (Fig. 1).

### Characterization of nanostructures

The nanostructures used in this study were characterized by transmission electron microscopy (TEM, Zeiss-EM10C-100 kV, Germany), Energy-dispersive X-ray spectroscopy (EDS) (FESEM, Sigma VP, ZEISS, Germany), and Fourier transform infrared (FTIR) spectrometer (Perkin Elmer, UK).

### Antibacterial function against *E. faecalis* biofilms

#### Chemo-mechanical preparation

Sample size was calculated according to Abbaszadegan et al. [31] study and the following formula [32]:

$$n = \frac{{\lambda_{g,\alpha ,1 - \beta } }}{\Delta }.$$

A total of 108 extracted human mandibular premolars with mature apices, which were extracted due to orthodontic or periodontal reasons were used in this study. The teeth were single-rooted, non-carious, and without fractures. The sample teeth were stored in distilled water to prevent probable dehydration till used. To achieve a standardized root length of 15 mm, all sample teeth were decoronated from 2 to 3 mm below the cementoenamel junction with a safe-sided diamond disc under water cooling. A #15 k-file (Dentsply, Maillefer, Ballaigues, Switzerland) was used to ensure the teeth had only one canal. The working length was obtained by subtracting one mm from the length measured when the tip of #15 k-file was first observed at the apical foramen. The root canals were prepared to the working length by sequential use of the ProTaper rotary system to file #F3 (Dentsply, Maillefer Tulsa, OK, USA). Then, they were irrigated with 2 ml of 2.5% NaOCl (Sigma-Aldrich Corporation, St. Louis, MO, USA) by using a plastic syringe with a 30-gauge needle (Cerkamed, Poland) between each instrument change.

To remove the smear layer, the root canal was consecutively rinsed with 5 ml of 17% ethylenediaminetetraacetic acid (EDTA) (Wizard; Rehber Chemical Co., Ltd, Istanbul, Turkey) and 5 ml of 5.25% NaOCl (Sigma-Aldrich Corporation, St Louis, MO, USA) for 5 min each (total = 10 min). To prevent bacterial leakage, the root canal apices were sealed with resin composite. Besides the coronal access cavity, the external root surfaces were sealed with nail polish to prevent bacteria leakage from the accessory lateral canals.

#### Root canal contamination

A culture with 96 wells was used to mount and fix specimens. Experimental samples (each with 10 samples) were randomly distributed into nine 96-well cell culture microplates and 6 control microplates (each containing three samples). They were all sterilized with ethylene oxide (Acecil, Campinas, São Paulo, Brazil). The root canals were filled with brain heart infusion (BHI) broth and their sterilization efficacy was evaluated. The samples were incubated (Mart Microbiology BV, the Netherlands) for 48 h at approximately 95% humidity and 37 °C, and microbial evaluation was done on the samples taken from each root canal.

Under a laminar flow chamber, the root specimens were inoculated. By using a 5-ml BHI broth media, the 2-day isolated pure culture colonies of *E. faecalis* (grown on BHI agar plates) were conceded in a 5-ml BHI broth medium. They were adjusted to achieve spectrophotometric turbidity of 1.5 × 108 CFU/ml. The root canals were contaminated under a laminar air-flow cabinet with 10 μl inoculums of *E. faecalis*. The samples were incubated at 37 °C for 3 weeks. Meanwhile, BHI was applied to the root canals (except for the negative control) on alternate days (QOD) by 0.5-ml insulin syringes to have continuous bacterial feeding. Gram staining and catalase reaction evaluations were conducted to evaluate the purity of the bacterial culture.

After 21 days, the primary microbial assessment was conducted by flooding the canal with sterile saline while inserting a Hedström file #30 into the canal for scraping the dentin throughout the procedure. Then, 3 sterile paper points (Gapadent Co. Ltd., Korea) were put in each canal for 60 s, withdrawn under laminar flow, and transferred into the tubes containing 1 ml of BHI. The tubes were vortexed for 60 s and the corollary solution was consecutively diluted tenfold in BHI broth. Aliquots of 100 μl of the suspension were smeared on BHI agar plates and incubated at 37 °C for 1 day. By using the CFU/ml counts of *E. faecalis*, bacterial growth was deliberated and validated with colony morphology and gram stain.

#### Intracanal medication

The root canals were re-prepared with 5 ml of sterile saline solution, followed by 17% EDTA, which lasted 3 min. The root canals were irrigated with sterile saline solution and dried with sterile paper points. The microplates containing the roots were randomly assigned to be medicated with one of the following intracanal medicaments as GO (n = 30), DAP (n = 30), GO-DAP (n = 30), positive control (sterile saline, n = 9), and negative control (no bacterial contamination, n = 9).

DAP was applied to the root canals by using size 30 spiral fillers (Dentsply; Mailer, Ballaigues, Switzerland) and condensed with hand pluggers (Dentsply India Pvt Ltd.; Mumbai, India). The graphene oxide was applied by a #30 K-file (Mani Inc.; Tochigi-Ken, Japan). Any excess medicament was eliminated and the access cavities were blocked by sterile cotton pellets. Each group was subdivided into three subgroups with respect to the exposure time of 24 h, 7 days, and 14 days. In other words, specimens were randomly allocated into 15 groups including 9 experimental (n = 10) and 3 control groups (n = 3).

The samples were incubated in a microaerophilic environment at 37 ℃ for the predetermined contact time defined for each subgroup. The medicaments were removed by using #30 K-file and rinsed with 5 ml of sterile saline. Except for the controls, the specimens were rinsed again with 2 ml of sterile saline. Microbiological harvests were conducted for the predetermined incubation duration with medicaments by using sterile Gates Glidden drills #5 (Mani Inc.; Tochigi-Ken, Japan). This sampling technique was adopted from previous studies [31, 33, 34]. To standardize dentinal shavings collection, roots with similar morphology and length were chosen. The sampling and preparation technique was identical in all experimental groups. The canals were drilled up to 10 mm of canal length 3 times, 10 s each. Dentin shavings were transferred into 1 ml of sterile BHI and vortexed for 60 s, followed by serial tenfold dilutions (up to 5 times) on BHI broth. Eventually, they were incubated in an anaerobic setting at 37 °C for 1 day. Aliquots of 100 μl from the suspensions were smeared on BHI agar plates, followed by incubation at 37 °C for a day. CFU/ml counts of *E. faecalis* were used for measuring bacterial growth.

### Statistical analysis

Statistical analyses were done with SPSS software (version 22, SPSS Inc., IL, USA). Given the abnormal distribution of data, intergroup and intragroup CFU/ml percentage reduction over different contact times were compared through the Kruskal–Wallis and Friedman tests, respectively. *P* < 0.05 was regarded as statistically significant.

## Results

### Characterization

**Fig. 2:**
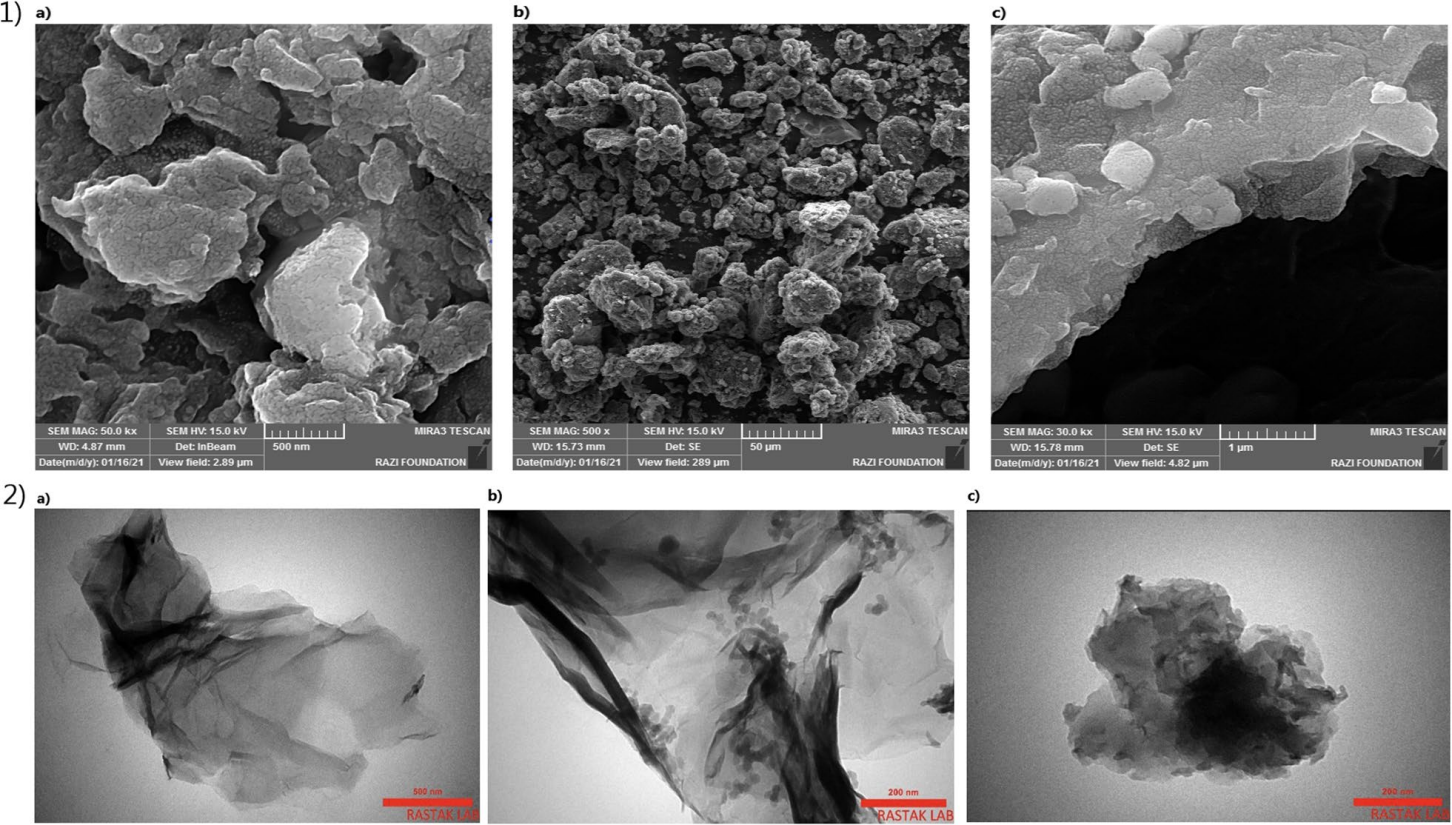
(1) SEM analysis of (a) GO, (b) DAP, (c) GO-DAP. (2) TEM images of (a) GO, (b) DAP, and (c) GO-DAP as antimicrobial agents

The morphology of GO, DAP, and GO-DAP was characterized through scanning electron microscopy (SEM) and TEM (Fig. 2). The loosely stacked and typical wrinkled structure of the GO and GO-DAP was observed in SEM micrographs. This wrinkled nature is essential to prevent collapse-back in a graphitic structure [35]. The wrinkled surface of GO and GO-DAP was also visible in TEM micrographs.

**Fig. 3:**
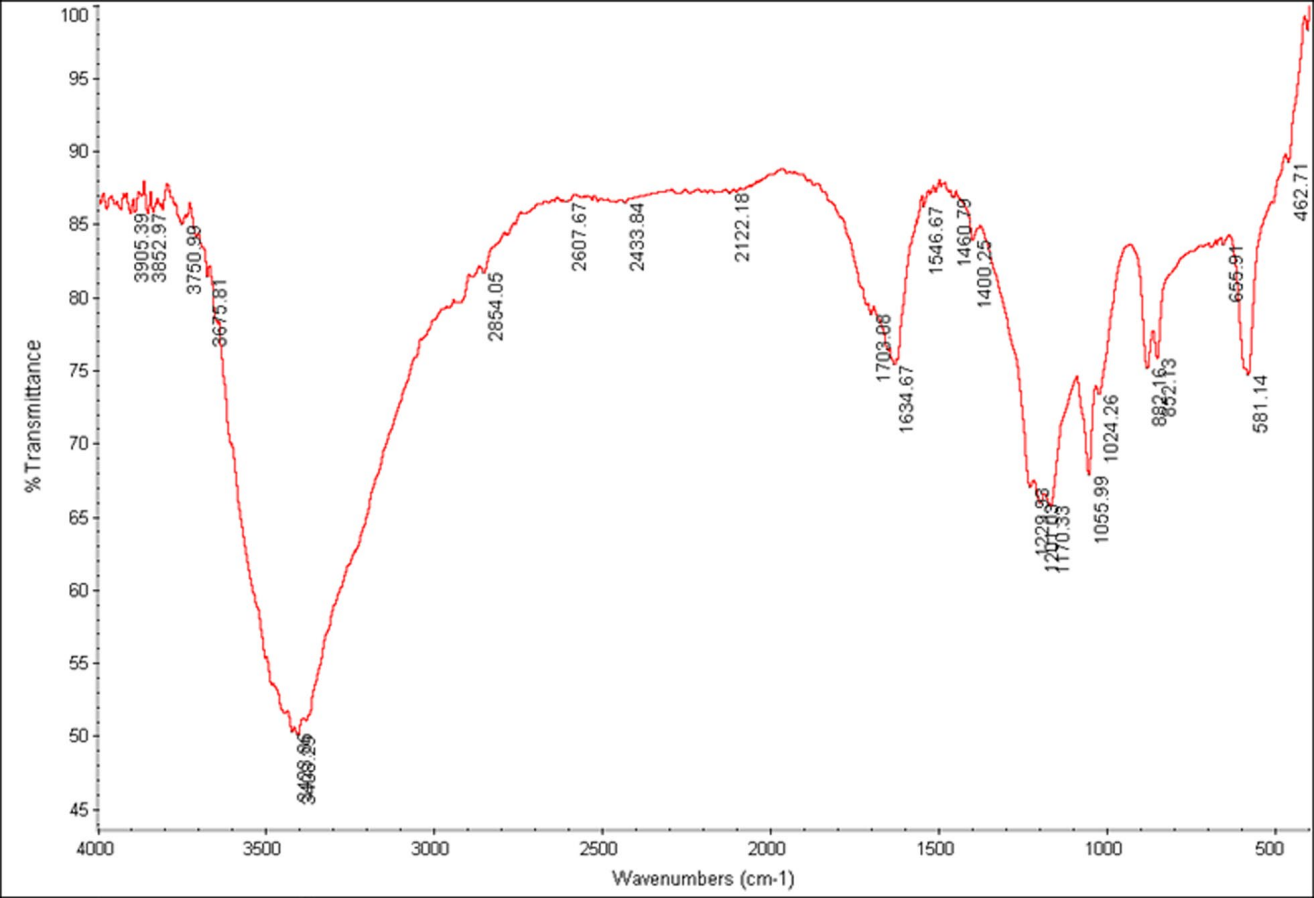
FTIR spectra of GO

**Fig. 4:**
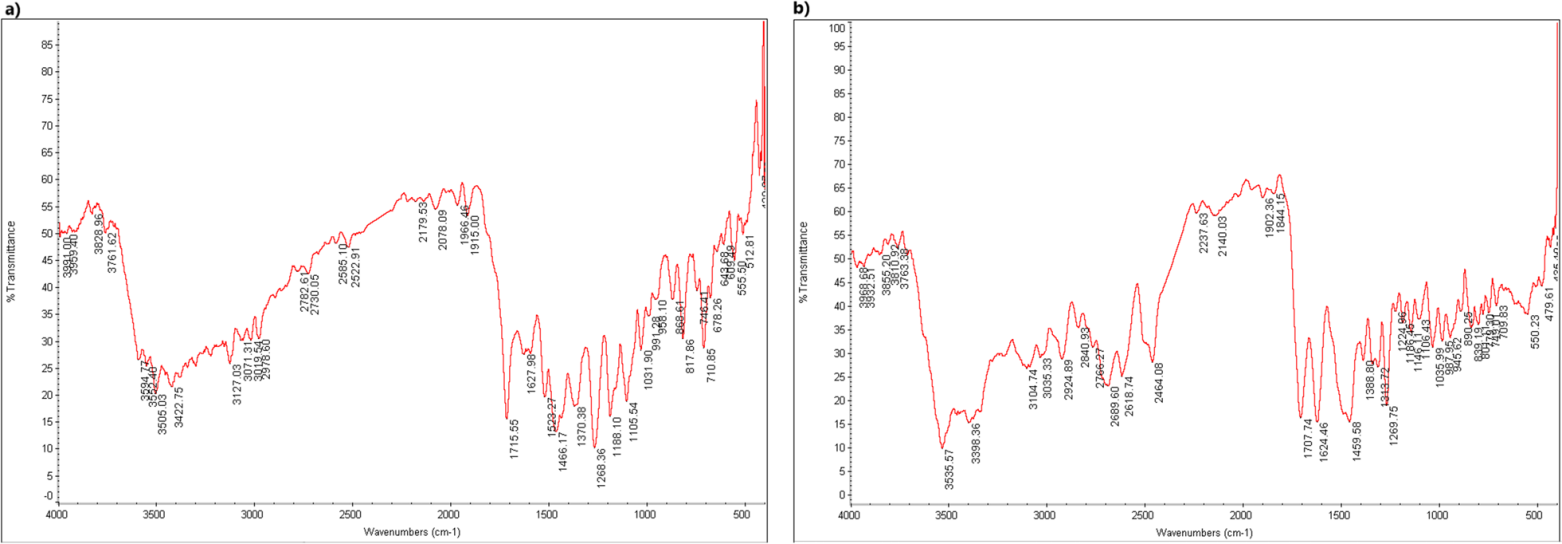
FTIR spectra of **a** GO-DAP, **b** DAP

Figure 3 demonstrates the FTIR spectra of the GO for the wavelength range of 450–4000 cm^−1^. The main characteristic peaks of the GO group were in good agreement with what was previously reported [36–39]. The carbonyl, alkoxy, and epoxy functional groups were confirmed by the FTIR peaks at 1703 cm^−1^, 1055 cm^−1^, and 1229 cm^−1^, respectively. The peaks at 3408 cm^−1^ and 1634 cm^−1^ were assigned to OH stretching vibration and C=C benzenoid vibration. Figure 4 displays the FTIR spectra of the DAP and GO-DAP groups. At the FTIR spectra of DAP, the characteristic peaks respectively contributed to NH stretching vibration (3398 cm^−1^), (CH) aromatic vibration (3035 cm^−1^), (CH) aliphatic stretching (2924 cm^−1^), C=C vibration (1624 cm^−1^), N=O (1452 cm^−1^), and ring torsion band (826 cm^−1^), which were in line with previous reports [40, 41].

### Antibacterial evaluation

Contamination of all specimens with *E. faecalis* was confirmed at the very first step of the experiment. The CFU/ml counts of the positive control samples revealed no statistically significant difference among the three studied contact times. Bacterial growth was denied in the negative control samples at the three studied time points. Table 1 summarizes the results of the descriptive analysis. The GO-DAP was the most efficient intracanal medicament against *E. faecalis* which completely eradicated this microorganism within 1 day. The results of the Kruskal–Wallis test revealed a significant difference in the CFU/ml counts among the experimental groups at all time points (*P* < 0.05). After 1 day and 7 days, GO-DAP remarkably eliminated higher bacterial count compared to GO and positive control (*P* < 0.05). Also, the reduction of bacterial count by DAP was significantly higher than positive control group after 1 day and 7 days (*P* = 0.027). After 14 days, GO-DAP and DAP resulted in a remarkable reduction of bacterial count compared to GO (*P* = 0.005 and *P* = 0.039, respectively).

**Table 1:** Intergroup comparative analysis showing the reduction percentage of colony-forming units (CFUs) after 24 h, 7 days, and 14 days of contact with intracanal medicaments on *Enterococcus faecalis* biofilms

Contact time	GO-DAP	DAP	GO	Positive control	*P* value
24 h	6.00	16.50	28.30	31.20	< 0.001
7 days	8.60	14.35	27.10	31.95	< 0.001
14 days	10.50	13.50	26.80	31.20	< 0.001

Values are presented as mean rank

*GO* Graphene oxide, *DAP* Double antibiotic paste

The bacterial count remarkably decreased from the initial sampling after 1 day to the last sampling after 14 days. Statistically significant reduction of CFU/ml counts after 1 day was only seen in the GO-DAP group (*P* = 0.002). At all allocated contact times, the GO-DAP and DAP showed superior antibacterial efficacy against *E. faecalis* than GO per se (*P* < 0.05). Intragroup comparisons revealed a significant reduction of CFU/ml count in GO, GO-DAP, and DAP groups after 7 and 14 days (*P* < 0.05).

## Discussion

For optimum use of biomaterials in endodontics, their antibacterial activity, biocompatibility, mechanical properties, sustainability, and shelf life should be considered [42, 43]. Given the excellent properties and favorable antibacterial activity of GO against different bacterial pathogens, the present study assessed the antibacterial efficacy of GO-DAP as a novel intracanal medicament against *E. faecalis*. The null hypothesis was rejected as a significant difference in the CFU/ml counts among the experimental groups was observed at all time points.

Three-week-old preformed biofilms of *E. faecalis*, facultative anaerobic gram-positive cocci, were chosen for the present study as they are the dominant pathogen in failed endodontic therapy [44]. Their unique features make them a highly resistant endopathogen that complicates endodontic therapy and necessitates the development of more efficient alternative irrigants and medicaments [45–47].

According to the present findings, the GO-DAP was the only agent that significantly reduced and entirely eradicated the *E. faecalis* bacterial load within 1 day (*P* = 0.002). Even the reduction of bacterial count with this agent was statistically higher than the DAP (*P* = 0.042). There is no exactly similar report in the literature the results of which can be directly compared with the present findings. However, some partly similar reports have stated that brief intracanal exposure to DAP was unable to completely disrupt *E. faecalis* [7, 48]. Unlike DAP, GO-DAP could completely eradicate *E. faecalis* within 24 h, which highlights the promising synergistic antibacterial efficacy of GO-DAP.

In the present study, the antibacterial efficacy of DAP against *E. faecalis* was higher than that of the GO at all the allocated contact times (*P* < 0.05). DAP reduced the initial bacterial count up to 98.22% in just 1 day and eradicated all the bacteria after 14 days. In accordance with the present findings, Sadek et al. [49] and Sabrah et al. [50] reported that DAP was a potent eradicator of viable bacteria counts (> 99%).

The antibacterial mechanism of GO is attributed to its particular two-dimensional structure that vigorously interacts with the bacterial lipid bilayer, separates the lipid molecules from the bacterial membrane, and, as a result, destroys it [51]. Nanda et al. [52] investigated the precise molecular mechanism of GO against *E. faecalis* by Raman spectroscopy. They concluded that GO degrades the inner and outer bacterial cell membrane of *E. faecalis*, thereby, Adenine and protein are released from the bacteria and it dies. Additionally, they reported that GO had an identical mechanism of antibacterial activity against both gram-positive and gram-negative microorganisms. It was also noted that increasing the GO concentration induced degradation of the inner and outer bacterial cell membranes of both gram-negative and gram-positive bacteria. In the current study, GO decreased the initial bacterial load by 42.78% within 24 h and 82.90% after one week. Complete eradication of *E. faecalis* was not achieved even after 2 weeks (97.54%).

The antibacterial efficacy of GO was significantly inferior to that of GO-DAP and DAP per se. To date, no study has evaluated the efficacy of GO as an intracanal medicament; although, few investigations have assessed the antibacterial efficacy of graphene or its derivatives against *E. faecalis* [26, 27, 53–55]. In accordance with the present findings, Ioannidis et al. [26] reported that Ag-GO reduced the bacterial load by 57%, being less efficient than NaOCl, the gold standard of endodontic irrigants. On the contrary, Sharma et al. [27] observed that the total microbial biovolume of Ag-GO nanoparticles was 86.85%, which was superior to NaOCl (80.40%), although it was statistically insignificant. However, two other studies declared that the incorporation of GO was promising in photodynamic therapy against *E. faecalis* and significantly decreased the bacteria count (up to 99.4%) [53, 54].

Another study stated that GO significantly decreased the bacterial load after 72 h of incubation [55]. Likewise, Mahmoud et al. [56] fabricated graphene quantum dots@gemifloxacin@hybrid double-layered Fe/Al, which effectively acted against *E. faecalis*. Furthermore, the minimum inhibitory concentration of cystamine-conjugated GO against *E. faecalis* revealed its strong antibacterial activity and great reactive oxygen species effects with low cytotoxicity [57]. Niranjan et al. [58] assessed the antibacterial activity of reduced graphene oxide (rGO) loaded with sulfur and sulfur-selenium nanoparticles. Their findings elucidated that, unlike rGO-S/Se NPs, the antibacterial efficacy of rGO and rGO-S was not favorable. Since the GO fight against *E. faecalis* is concentration- and time-dependent, the contrasting findings of the aforementioned studies could be attributed to the different concentrations of GO or its time of contact with the root canal system [59]. Additionally, medium culture conditions and the purity of GO may affect its antibacterial activity [60]. Another influencing factor of the antibacterial activity of nanoparticles is their size, which may lead to differences between studies [58].

## Conclusion

Within the limitations of the present study, GO-DAP revealed antibacterial efficacy against *E. faecalis* even after 24 h and represented a promising candidate as an intracanal medicament in root canal therapy.

## Data Availability

The datasets used and/or analyzed during the current study are available from the corresponding author upon reasonable request.

## Abbreviations

*E. faecalis*: *Enterococcus faecalis*
TAP: Triple antibiotic paste
DAP: Double antibiotic paste
GO: Graphene oxide
CFU: Colony forming unit
TEM: Transmission electron microscopy
EDS: Energy-dispersive X-ray spectroscopy
FTIR: Fourier transform infrared
EDTA: Ethylenediaminetetraacetic acid
rGO: Reduced graphene oxide
H_3_PO_4_: Phosphoric acid
H_2_SO_4_: Sulfuric acid
KMnO_4_: Potassium permanganate
H_2_O_2_: Hydrogen peroxide
